# Signature of climate dynamics on hydrological drought dynamics: A qualitative analysis

**DOI:** 10.1016/j.heliyon.2024.e39822

**Published:** 2024-10-26

**Authors:** Louis Kongoda Lisika, Jacques Celestin Moliba Bankanza, Louis Efoto Eale, Petrus Bompere Lemo, Jean Kigotsi Kasereka, Jean-Robert Bwangoy Bankanza, Vincent Lukanda Mwamba

**Affiliations:** a Mention Physique et Technologie, Faculté des Sciences et Technologies, Université de Kinshasa, B.P. 127, KinshasaXI, Kinshasa, Congo; b Faculté de Pétrole, Gaz et Energies nouvelles, Université de Kinshasa, B.P. 127, KinshasaXI, Kinshasa, Congo; c Laboratoire d’écologie politique (LAECOPOL), Université de Kinshasa, B.P. 127, KinshasaXI, Kinshasa, Congo; d Comité National de Protection contre les Rayonnements Ionisants (CNPRI), Kinshasa, DRC. B.P. 127, KinshasaXI, Kinshasa, Congo; e Department of Natural Resource Management, Faculty of Agronomy, University of Kinshasa, B.P.127, Kinshasa XI, Kinshasa, Congo

**Keywords:** Bayesian approach, Changepoint, Climate disturbance signal, Climate dynamics, Hydrological drought dynamics, Transient dynamics, Congo watershed

## Abstract

Understanding the complexity of climate dynamics is of paramount importance, because climate variability and change significantly affect water cycles and ecosystems. However, recurrent hydrological droughts that have been observed every 10 years on the two primary tributaries of the Congo River (the Ubangui and Kasaï Rivers) since the advent of the 1969 drought lack a plausible explanation for variability or climate change. This study proposes a plausible explanation for recurring hydrological droughts. Given the low rate of human activity and vegetation cover evolution in the Congo Watershed, we propose that climate dynamics play a crucial role in hydrological drought dynamics. By applying the Bayesian approach to a gridded precipitation database, we obtained posterior probability maps for each annual time step during our observational period (1940–2020). This provided a spatiotemporal representation of the areas affected by climatic disturbances, unlike previous studies that were limited to a spatial representation of the temporal location of the disturbances. Our qualitative analysis of the maximum intensity of the climate disturbance signal (CPS) revealed an average cycle of 10 years and eight months of signal migration. However, we observed that every 10 years since the advent of the drought during 1969, the hydrological drought occurrence dates coincided with CPS migration dates. This highlights the influence of this cycle on the hydrological drought dynamics. Nevertheless, because of the monthly scale involved in the propagation time from meteorological to hydrological drought, the error in this cycle was considered to be the propagation time of disturbances. Therefore, we recommend that future research should focus on estimating this time to test this hypothesis. This study underscores the significance of the cyclic dynamics of lengthy transient processes in understanding hydrological drought dynamics.

## Highlights

• The disturbances were more pronounced in the South than in the north of the basin.
• The climate of the Congo Basin was disturbed for half a century (1946–1997).
• 1946–1997 has been identified as a transitional period of the stable climate variation.
• The transitional period was characterized by a cyclic dynamic of 10 years ± 8 months.
• Cyclical dynamics modulated the dynamics of hydrological droughts.

## Graphical abstract

**Figure:**
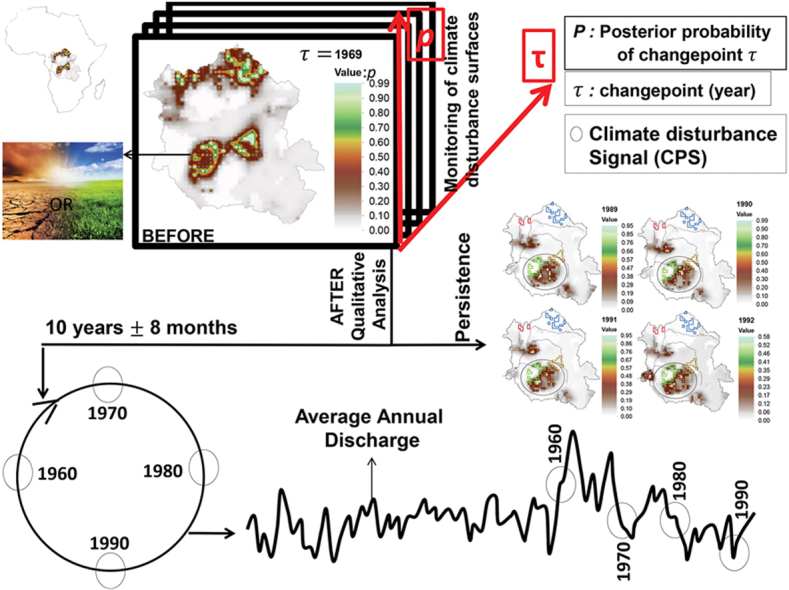

## 1 Introduction

Climate variability and change have several consequences on the water cycle and ecosystems, as indicated by the studies conducted by Sonwa et al. [1] and Zhou et al. [2]. Understanding climate dynamics can provide insights into the changes that affect ecosystems as well as surface and groundwater resources.

The Congo Watershed, which has significant ecological biodiversity [3,4] and water resources [5], is a critical wetland and one of the most exposed regions in the world, making it susceptible to climatic variations [[6], [7], [8], [9]]. Notably, the persistent and severe drought identified in 1969 resulted from climatic disturbance that led to a reduction in precipitation in this basin [[10], [11], [12]]. This decline in rainfall has repercussions that extend beyond precipitation, affecting hydrological systems (such as surface runoff, water levels, and flow) and ecosystems. This decrease in rainfall has been observed not only in the Congo Watershed but also throughout West Africa [[13], [14], [15], [16]]. If this reduction in precipitation endures longer in the Congo Watershed, it could induce desertification across the entire African continent [17].

The Congo Watershed serves as an eco-climatic shield that safeguards Africa from encroachment and aridification of the entire continent. Africa is predominantly a dry continent, with the Congo Watershed and Gulf of Guinea being the only wetlands on the continent, aside from Madagascar. The northern and southern regions of these wetlands comprise savannahs and steppes but also include extensive deserts, such as the Sahara to the north and the Kalahari to the south. Aridification in the north and ongoing expansion of the Sahara towards the south are causes of concern, as noted by Giannini et al. [17]. Certain regions of the Gulf of Guinea, such as Atakora in southern Benin [18], have been affected. However, Kalahari remains a desert, with its northern limits extending south of Angola [19]. The town of Moçamedes (Namibe) in Angola experiences low rainfall and is thus arid. Therefore, research focusing on climate variability or changes at the Congo Watershed scale is of significant interest on a continental scale in Africa.

The climatic disturbance that occurred in 1969 (or 1970) resulted in a substantial decrease in surface runoff, which was almost double that of rainfall [10,12,20,21]. This emphasizes the necessity of examining the dynamics of this disturbance within the watershed and its impact on both ecosystems and hydrological systems. However, these problems have received little attention in climate-variability research.

Ndehedehea et al. [6,7] showed that, on several scales, interactions between oceanic and atmospheric phenomena have strongly influenced moisture flux, surface runoff, such as the flow of the Congo River, and hydroclimatic extremes, notably droughts. The origin of the abnormally dry periods was attributed to sea surface temperature variations over the Indo-Pacific, associated with the strengthened tropical Walker circulation extending westward [22]. Nevertheless, a reduction in the flow of moisture from the ocean (linked to the flow of evaporation on the continent) has contributed to the occurrence of droughts [23] in the Congo Watershed. Furthermore, Orange et al. [24] showed a three-year time lag between the decrease in rainfall and the decrease in flow in the Ubangui sub basin, leading to a more pronounced lowering of the water table to the north of the region than to the south [25] from 1969 to 1994. This decrease in rainfall, estimated at 13 %, led to a persistent decline in flow of approximately 30 %, resulting in pronounced low water levels in this sub basin [20,26].

However, the increase in cultivated areas, representing only 3 % of the Ubangui sub basin, led Nguimalet and Orange [20] to conclude that the determining factor was climate variability, as emphasized by other authors [27,28]. To support these studies on climatic variability on any temporal scale, Cook and Vizy [29] proposed a scheme of hydrodynamic processes for regional and seasonal rainfall variations in the Congo Watershed. In addition, long-term trends in precipitation, droughts, and floods, and their future projections have been studied in this basin (Mabrouk et al. [8] and Karam et al. [30]). Mabrouk et al. [8] identified three climatic zones in the Congo Watershed. These three zones were characterized by negative seasonal and annual trends in rainfall and drought, underlining the increased risk of drought in the Congo Basin, with the exception of a small zone located in the south of the Congo Watershed. In the future, the Congo Watershed will experience more frequent droughts and floods in response to climate change [30].

This decrease in rainfall has affected not only the surface and groundwater resources in the Congo Watershed but also the entire region, its water cycle [1], and ecosystems [2], thus endangering populations that depend on the services they provide [31,32]. However, research in the Congo Watershed has focused primarily on long-term fluctuations in surface and groundwater resources in response to climatic fluctuations and oceanic–atmospheric interactions. Although a schema of rainfall variability dynamics has been proposed on a seasonal scale [29], no study has proposed a schema on the annual and decadal scales for the Congo Watershed.

The variation in water levels in various watercourses and the retreat or advance of riverbeds, whether sudden or slow, random, or significant in relation to rainfall dynamics, has not been clearly addressed in these studies. The same applies to various ecosystem changes, particularly in marginal areas. However, abnormally long seasons (the dry season of the last three decades of the last century) and/or more frequent intense rainfall have caused significant damage to ecosystems [2,31,32]. In poor countries, such as the Democratic Republic of the Congo (DRC), which does not have the means to adapt to these new conditions, analysis of the dependence between climatic dynamics and hydrological and ecological responses will contribute significantly to mitigating the adverse effects of climate change.

The hydrological drought observed in the Congo River and its tributaries has led to research focusing on its possible causes [22]. However, every 10 years in the early 1970s, 1982, and the 1990s [10,12,21,26], the surface runoff decreased to its lowest level. No plausible explanation for this decline has been proposed.

However, natural changes in climatic processes, such as abrupt changes in precipitation, can be characterized by a change point, otherwise known as a point reflecting short- or long-term climatic persistence [33,34]. A change point, defined as an abrupt change in the distribution parameters of a dataset, occurs when the data are separated into two subsets with different statistical properties, such as changes in the central tendency or scale [33].

Several methods [35] have been developed and successfully used to detect one or more change points. These can be grouped into two categories: parametric and non-parametric methods. For example, Pettitt's method [36] is non-parametric, unlike the parametric method of Lee and Heghinian [37]. These two approaches differ in the assumption of a particular statistical distribution imposed by parametric approaches in contrast to non-parametric approaches, although both approaches always assume a distribution for the models used [33]. The parametric approach assumes the a priori existence of a point somewhere in a time series and provides a posterior probability of change point (probability) at each time step [37,38].

We employed the Bayesian parametric method of Lee and Heghinian [37] in the current study because it provides the probable moment of detection of a changepoint, unlike the non-parametric approach, in particular, the Pettitt [36] method, which provides the probability that the hypothesis of detection is true. Therefore, the Lee and Heghinian [37] method can be considered a detector that is sensitive to a changepoint. This allows for a two-dimensional representation (sensitivity and location) of a changepoint, unlike the Pettitt [36] method which can only provide a representation of the temporal location of a detected changepoint. In addition, the selection of the Lee and Heghinian method [37] was based on its simplicity of implementation and sensitivity in detecting only one changepoint in a time series. Lee and Heghinian [37] used this Bayesian parametric approach to determine the marginal and joint posterior distributions of the change point in central tendency and scale. The Lee and Heghinian method was then applied by several authors to detect this point, such as Laraque et al. [10] on the right bank of the Congo Watershed and Servat et al. [39] in non-Sahelian West and Central Africa.

Although it is possible to estimate the probability that a detection hypothesis is true in a non-parametric approach as well as the probable moment of detection of a change point in a parametric approach, point analysis is often limited to the spatial distribution of their temporal locations. Servat et al. [39] presented a classification map for these points in non-Sahelian West and Central Africa. Motivated by the response of hydrological systems to climate variability, several authors (Moukandi N'kaya et al. [12]) have estimated the average rainfall in hydrological units. This made it possible to estimate the temporal location of a change point in a selected hydrological unit. Since these units are static in space, so are the temporal locations of these points.

Although the subdivision of a region into different hydrological units can also consider the spatial heterogeneity of a change point [10,12], it is not possible to interpolate the flow from a point to a nearby non-riparian region. This is explained by the fact that the discharge at a point is punctual and, therefore, excluded from any spatial variability. Consequently, to better express ecosystem sensitivity to spatial variations in climatic conditions, it is preferable to use climatic units rather than hydrological units [8,40,41].

Climatic data grids or gauge data from different meteorological stations provide an excellent framework for delineating units that are sensitive to dynamics and monitoring areas affected by climatic disturbances or climate change. Therefore, this study used the Climate Research Unit (CRU) gridded precipitation database, version TS 4.05 to delineate climatic units. Note, however, that this database is considered representative of the particular characteristics of precipitation in an area of 0.5 × 0.5°dec.

However, to consider the particular characteristics of spatial and temporal heterogeneity of climatic disturbance, this study used the posterior probabilities of changepoints instead of the temporal location of changepoints on different hydrological units, considering that this point is detected if, and only if, its probability is ≥ 0.5. This results in a spatial distribution of posterior probabilities at each time step, which allows spatiotemporal representation of the areas affected by climatic disturbance. This representation allowed us to introduce the concept of climate disturbance signal, the latter of which is defined by the posterior probability of the change point. Therefore, the classification of the values of this posterior probability makes it possible to define the signal intensities. Servat et al. [39] presented such a representation using a pluviometric index instead of probability at 10-year time intervals, from 1950 to 1980.

Inspired by the aforementioned explanations, this study proposes an explanation for the dynamics of recurrent hydrological droughts observed every 10 years on the two primary tributaries of the Congo River (the Ubangui and Kasaï Rivers). Applying the Lee and Heghinian [37] method based on the CRU TS 4.05 precipitation data at 0.5 × 0.5°dec at the Congo Watershed scale, this study provides: (1) a simple and excellent tool for delineating and monitoring the areas affected by climatic disturbances, and (2) a proof of climate dynamic effects on hydrological drought dynamics.

## 2 Materials and methods

### 2.1 Study area and data

**Fig. 1:**
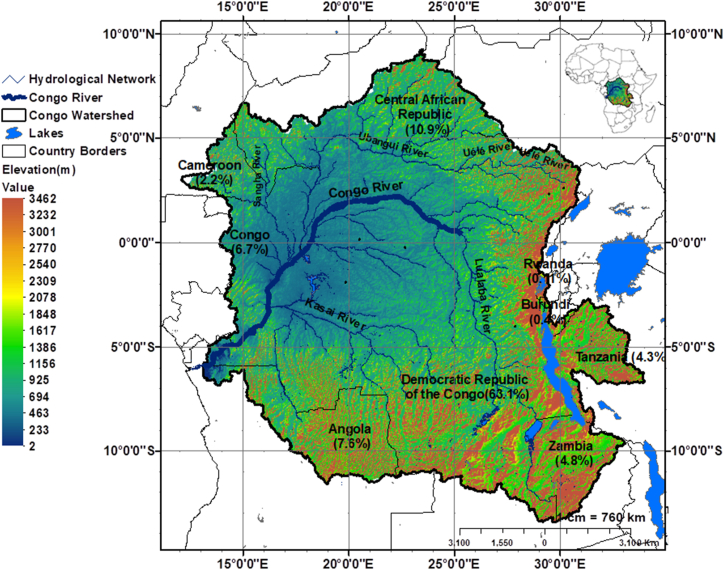
Location of the Congo watershed on the African continent.

The Congo Watershed is located at the heart of Africa (Fig. 1). It has an area of approximately 369 2792 ${\text{km}}^{2}$ spanning 09°20′N–13°35′S and 12°05′E−34°00′E. The Congo Basin is located in the DRC and accounts for approximately 63 % of the total area. The remainder of the area is distributed among Cameroon (2.2 %), the Central African Republic (10.9 %), Angola (7.6 %), Burundi (0.4 %), Congo (6.7 %), Tanzania (4.3 %), Zambia (4.8 %), and Rwanda (0.11 %).

The Congo River is the second largest river in the world [42], both by its annual modulus estimated at 41 000 m^3^ s^−1^ and by the size of its watershed [42,43]. It is the only African river to have a dense hydrographic network. In addition, it is also characterized by a length of 4.700 km and a very low general slope of 0.033 %, with an irregular evolution from upstream to downstream [42]. The primary navigable tributaries of the river are the Luapula, Lualaba, Lomami, Ruki-Tshuapa, Ubangui, Sangha, and Kasaï River. The primary tributaries that feed the river are the Kasaï, Ubangui, and Sangha rivers [42]. The basin on both sides of the equator provides a river with a regular and stable bimodal hydrological regime [42]. A depression that does not exceed 400 m in altitude dominates the basin center. It primarily comprises sandy sandstone formations and Mesozoic argillite topped by ferralitic soil. This depression is covered by dense rainforests, and 35 % of the basin area is partially flooded during floods [43]. The Congo Watershed is subdivided into the following climatic zones: (1) the equatorial zone located at the center and astride the equator, which is characterized by the absence of a true dry season; (2) the tropical zone to the north and south of the equatorial zone; and (3) the temperate zone over the mountains in the east [44]. In the equatorial zone of the Congo Watershed, annual precipitation varies between 1500 and 2000 mm, and the average temperature is estimated to be 26 °C [42].

The Congo Watershed's varying characteristics give it enormous potential for the development of its water resources on a regional scale, such as hydropower, irrigation, and navigation.

In this study, we used the CRU TS 4.05 gridded dataset provided by the CRU of the University of East Anglia. CRU uses an iterative homogenization procedure to obtain the homogenized data. Based on this procedure, a reference series was used to correct for any heterogeneity in station records. The corrected data were then merged with an existing database and converted into anomalies [45]. The resulting anomalies were then interpolated to produce gridded data with a 0.5 × 0.5°dec spatial resolution using the spline technique and inverse weighted distance functions. Both techniques are adapted to irregularly distributed data in space. The dataset used in this study comprised CRU TS 4.05 gridded monthly precipitation with a spatial resolution of 0.5 × 0.5°dec for 1940–2020, covering the entire area of the Congo Basin, accounting for 1192 nodal points. The CRU TS 4.05 dataset was described in detail by Harris et al. [45].

### 2.2 Methods

The general methodology of this study consisted of applying the Bayesian parametric method of Lee and Heghinian [37] to the monthly precipitation grid CRU TS 4.05 database which covers 1901–2020 on a global scale. Based on this general methodology, the following specific methods are used: (1) spatiotemporal representation of the Bayesian parametric method of Lee and Heghinian [37]; (2) selection of the time window of this spatiotemporal representation; (3) grouping classification of changepoints detected in different regions in the selected time window, and (4) estimation of the persistence of these different regions.

#### 2.2.1 Bayesian parametric approach of Lee and Heghinian

Lee and Heghinian [37] proposed a hypothesis in which no information is available a priori on a series of n values of random variable $x_{i}$ corresponding to the observations for different time steps (i = 1, …, n). They also assumed that the random series is composed of two parts ($x_{1}$, $x_{2}$, …, $x_{\tau }$) and ($x_{\tau +1}$, …, $x_{n}$), whose respective means are μ and μ+δ. The primary objective of this approach is to estimate τ and δ, which represent the changepoint location and deviation amplitude of a changepoint (the amount shift), respectively. To achieve this, Lee and Heghinian [37] proposed a model, whose mathematical expression is presented in Equation (1). They also assumed that there was no change in variance in this model.

(1) $$x_{i}=\left\{ \begin{array}{c} \mu +\varepsilon_{i},\\ \mu +\delta +\varepsilon_{i}, \end{array}\right.\;\begin{array}{l} \;i=1.2,\ldots ,\tau \\ \;i=\tau +1,\ldots ,n \end{array}$$

where $\varepsilon_{i}$, (i = 1, …, n) are independent and typically distributed with zero mean and constant variance $\sigma^{2}$ et, and τ and δ are the model parameters.

By making certain assumptions about the non-informative distributions of the a priori independent variables of Model (1), Lee and Heghinian [37] estimated f (τ |$x_{1}$, $x_{2}$, …, $x_{n}$), the posterior distribution of τ. This distribution is given by Equation (2) where R(τ) is given by relation (3). Equations (4), (5), (6) represent respectively the mean of: τ first observations (equation (4)), n-τ last observations (equation (5)) and n observations of the entire series (equation (6)). However, the estimation of the mean of the first three and last three values of a time series uses a limited number of observations and should be taken with caution from the analysis of the relationship results (2).

(2) $$f\left(\tau |x_{1},x_{2},\ldots ,x_{n}\right)\approx {\left[\frac{n}{\tau \left(n-\tau \right)}\right]}^{1/2}{\left[R\left(\tau \right)\right]}^{-\left(n-2\right)/2},\tau =1.2,\ldots \left(n-1\right)$$

where:

(3) $$R\left(\tau \right)=\left[\sum_{i=1}^{\tau }{\left(x_{i}-{\bar{x}}_{\tau }\right)}^{2}+\sum_{i=\tau +1}^{n}{\left(x_{i}-{\bar{x}}_{n-\tau \;}\right)}^{2}\;\right]{\left[\sum_{i=1}^{n}{\left(x_{i}-{\bar{x}}_{n}\right)}^{2}\;\right]}^{-1}$$

with:

(4) $${\bar{x}}_{\tau }=\tau^{-1}\sum_{i=1}^{\tau }x_{i}$$

(5) $${\bar{x}}_{n-\tau \;}={\left(n-\tau \right)}^{-1}\sum_{i=\tau +1}^{n}x_{i}$$

(6) $${\bar{x}}_{n}=\frac{1}{n-1}\sum_{i=1}^{n}x_{i}$$

The authors also demonstrated that the posterior distribution of δ given τ, f (δ |τ, $x_{1}$, $x_{2}$, …, $x_{n}$), is an uncentred Student's t distribution with (n-2) degrees of freedom, with mean $\mu_{\tau }\left(\delta \right)$ and variance $\sigma_{\tau }^{2}\left(\delta \right)$. The mathematical expressions of the last two are given by Equations (7), (8), respectively:

(7) $${\hat{\mu }}_{\tau }\left(\delta \;\right)\approx {\bar{x}}_{n-\tau }-{\bar{x}}_{\tau }$$

(8) $${\hat{\sigma }}_{\tau }^{2}\left(\delta \right)\approx \frac{\text{nR}\left(\delta \right)}{\tau \left(n-\tau \right)\left(n-2\right)}\sum_{i=1}^{n}{\left(x_{i}-{\bar{x}}_{n}\right)}^{2}$$

Epstein [46] estimated the function f (δ |τ, $x_{1}$, $x_{2}$, …, $x_{n}$) of δ, given by equation (9),

(9) $$f\left(\delta |\tau ,x_{1},x_{2},\ldots ,x_{n}\right)=\frac{{\left(n-2\right)}^{-\frac{1}{2}}}{\sigma_{\tau }\left(\delta \right)B\left(\frac{1}{2},\frac{n-2}{2}\right)}{\left[1+\frac{\left(\delta -\mu_{\tau }\left(\delta \right)\right)}{\left(n-2\right)\sigma_{\tau }^{2}\left(\delta \right)}\right]}^{-\frac{\left(n-1\right)}{2}}$$

where:

(10) $$B\left(x,y\right)=\frac{\Gamma \left(x\right)\Gamma \left(y\right)}{\Gamma \left(x+y\right)}$$

(11) Γ(x)=(x−1)!

Equations (10), (11) represent the beta and gamma functions, respectively: The estimates of the mean and variance of distribution f (δ |τ, $x_{1}$, $x_{2}$, …, $x_{n}$) are given by Equations (7), (8), respectively.

Lee and Heghinian [37] also demonstrated that the posterior distribution of δ (the amount shift) f (δ |, $x_{1}$, $x_{2}$, …, $x_{n}$) is given by Equation (12). The latter is considered the average of the distribution of τ weighted by the posterior distribution of δ, given τ.

(12) $$f\left(\delta |x_{1},x_{2},\ldots ,x_{n}\right)=\sum_{\tau =1}^{n-1}f\left(\delta |\tau ,x_{1},x_{2},\ldots ,x_{n}\right).\;f\left(\tau |x_{1},x_{2},\ldots ,x_{n}\right)$$

The level of significance of the amount of shift can be estimated using the Bayesian credibility interval C (1-α) (δ) = [a,b], which represents the values that this variable can take at a credibility level of 100 (1 - α)% [47]. Berger [48] proposed a mathematical expression for this interval given by Equation (13), where Prob denotes the posterior probability.

(13) $$\text{Prob}\left\{a\leq \delta \leq b\right\}=\int_{a}^{b}f\left(\delta |\tau ,x_{1},x_{2},\ldots ,x_{n}\right)d\delta =1-\alpha$$

where:

(14) $$\text{Prob}\left\{\delta \leq a\right\}=\text{Prb}\left\{\delta \geq b\right\}=\frac{\alpha }{2}$$

A specific value $\delta_{0}$ of δ can be considered plausible if $\delta_{0}$ ϵ C while roughly representing the distribution f (δ |τ, $x_{1}$, $x_{2}$, …, $x_{n}$) by Bayesian credibility interval $C_{1-\alpha }\left(\delta \right)$. This makes it possible to examine whether the value $\delta_{0}$ = 0, which represents the hypothesis of no change in the mean, is credible by examining whether the value $\delta_{0}$ belongs to the interval $C_{1-\alpha }\left(\delta \right)$ [47]. Bernier [49] generalized the Bayesian approach of Lee and Heghinian (1977) using an a priori conjugate distribution with additional parameters.

#### 2.2.2 Spatiotemporal representation of the Lee and Heghinian Bayesian parametric approach

The methodological approach of this study consisted of applying the Bayesian parametric approach of Lee and Heghinian [37] to the monthly precipitation grid CRU TS 4.05 database, which covers a global scale from 1901 to 2020. However, to limit ourselves to our study area and annual time scale, this monthly database was extracted on the Congo Watershed scale and transformed into a grid of annual total precipitation. The application of this approach by Lee and Heghinian [37] to the transformed grid of annual precipitation totals makes it possible to obtain a spatiotemporal representation of the posterior probability of the change points observed over the study period (1940–2020) on the Congo Watershed scale. However, a threshold fixed at 0.5 of the maximum values of the posterior probability is defined in the interval [0.5, 1.0], which allows for change point detection.

Let the matrix $x_{\left(i,j\right)}^{k}$ consist of a chronological series of annual precipitation totals, where i = 1, …m, j = 1, …m, and k = 1, …n represent latitude, longitude, and time, respectively. where m = 1192 is the total number of elementary cells with 0.5 × 0.5°dec resolution on the Congo Watershed scale, and n = 81 is the length of the observational period covering 1940–2020. The application of the Bayesian approach of Lee and Heghinian [37] to the matrix $x_{\left(i,j\right)}^{k}$ allows us to obtain two matrices $P_{\left(i,j\right)}^{\left(k|x^{k}\right)}=f_{\left(i,j\right)}^{\left(\tau |x^{1},x^{2},x^{3},{\ldots x}^{n}\right)}$ and $\Delta_{\left(i,j\right)}^{\left(q|\lambda^{q}\right)}=f_{\left(i,j\right)}^{\left(\delta |x^{1},x^{2},x^{3},{\ldots x}^{n}\right)}$. $P_{\left(i,j\right)}^{\left(k|x^{k}\right)}$ is the posterior distribution of change point k over the observational period and $\Delta_{\left(i,j\right)}^{\left(q|\lambda^{q}\right)}$ is the posterior distribution of the amount shift $\lambda^{q}$ on the Bayesian credibility interval $\left[\lambda^{r},\lambda^{s}\right]$)(q∈[r,s]). The two parameters τ and δ of model (1) respectively represent the kth changepoint corresponding to the mode of the posterior distribution of τ over the observational period and the amount shift (q∈[r,s]) corresponding to the maximum value of the posterior distribution of δ on the Bayesian credibility interval $\left[\lambda^{r},\lambda^{s}\right]$ ∀ i,j. The spatial representation is as follows: (1) $P_{\left(i,j\right)}^{\left(1|x^{1}\right)}$, $P_{\left(i,j\right)}^{\left(2|x^{2}\right)}$, $P_{\left(i,j\right)}^{\left(3|x^{3}\right)}$, …, $P_{\left(i,j\right)}^{\left(\tau |x^{\tau }\right)}$, …, $P_{\left(i,j\right)}^{\left(n|x^{n}\right)}$ at each time step k, and (2) $\Delta_{\left(i,j\right)}^{\left(q|\lambda^{q}=\delta \right)}$ on the Congo Watershed scale.

#### 2.2.3 Selection of the time window

The selection of the time window for the posterior probability of the changepoints over the observational period was made possible because of the density function of the detected changepoints. This function estimates the occupation density of a detected change-point value on the Congo Watershed scale. The time window of the spatial representation of the posterior probability is then selected by the detected change point, which corresponds to the maximum value of the density function of the change points detected on the Congo Watershed scale.

Let φ be a set composed of τ such that $P_{\left(i,j\right)}^{\left(k|x^{k}\right)}$ = $P_{\left(i,j\right)}^{\left(\tau |x^{\tau }\right)}$ , and define a density function γ with a value in φ as follows:

(15) $$\gamma =\frac{⋕\varphi }{m}\times 100$$

where ⋕φ is the total number of elements in set φ and m is the number of elementary cells on a regional scale, in our case, on the Congo Watershed scale. The value of τ taken from set φ which corresponds to the maximum value of the γ function, defines the time window of the spatial representation of the posterior probability of the change points over the observational period.

#### 2.2.4 Detected changepoint grouping classifications

The spatial representation of the posterior probability of the selected time window makes it possible to visualize the appearance of groupings with values ≥ 0.50. Using a visualization of the geographical locations of these groupings, we classified them into different geographical regions to properly locate the different areas, which were subdivided into different sub basins. We then extracted the posterior probability of the change points observed over the study period from the scales of the latter values.

#### 2.2.5 Changepoint persistence

The last methodological step consisted of applying the Pearson correlation [50] to the values of the posterior probability at each observed changepoint over the study period, with values of the posterior probability to the time window selected on the grouping regional scale of the detected changepoints. The objective of this last step was to estimate the persistence of a change point using Pearson correlation [50]. An interval of Pearson's correlation coefficient values (persistent interval) was defined to delimit the persistent period. In this study, the persistent interval of the Pearson correlation coefficient values was set to the interval [0.62, 1.0]. However, the selection of the persistence period was based on two criteria: (1) the selection of at least three consecutive Pearson correlation coefficient values belonging to the persistent interval, and (2) if there is a missing value between the two groups of values selected in step (1), the date corresponding to this missing value will be considered in the selection of the persistent period.

The persistence of a phenomenon can be defined as its similarity over time [51] and is characterized by a temporal correlation [52]. Several temporal correlations can be used to measure persistence, such as Pearson [50], Spearman [53] and Kendall [54]. These three coefficients are commonly used [[55], [56], [57], [58]]. However, Pearson's approach is parametric, unlike the other two approaches, which are non-parametric [55]. Spearman's correlation can be thought of as an application of Pearson's correlation on the data transformed in such a way as to satisfy the normal distribution assumption [56,57], whereas Kendall's correlation represents a probability, that is, the difference between the probability that the observed data is or is not in the same order [54]. Moreover, Spearman's correlation represents an adaptation of Pearson's correlation between two variables that cannot be measured quantitatively. Nevertheless, the Pearson's correlation is sensitive to outliers [55,58]. Croux and Dehon [59] concluded that Kendall's coefficient has a slight advantage over Spearman's coefficient, because its distribution quickly converges to a normal distribution. Among the three measures of similarity, we preferred to use Pearson's correlation because of its quantitative measurement sensitivity to the outliers. In this study, the Pearson correlation coefficient measures the similarity between $P_{\left(i,j\right)}^{\left(k|x^{k}\right)}$ at different observational time steps and $P_{\left(i,j\right)}^{\left(\tau |x^{\tau }\right)}$ at the time window selected on a geographical regional scale. Pearson correlation was defined as the covariate of the two variables divided by the product of their respective standard deviations [50]. The relationship below presents the mathematical formulation of the Pearson's coefficients. Let $P_{\left(i,j\right)}^{\left(k|x^{k}\right)}$ and ${\;P}_{\left(i,j\right)}^{\left(\tau |x^{\tau }\right)}$) be the Pearson correlation coefficients, denoted by $\rho^{k}$ and defined as follows:

(16) $$\rho^{k}=\frac{\sum_{i,j=1}^{m}\left[P_{\left(i,j\right)}^{\left(k|x^{k}\right)}-\bar{P_{\left(i,j\right)}^{\left(k|x^{k}\right)}}\right]\left[P_{\left(i,j\right)}^{\left(\tau |x^{\tau }\right)}-\bar{P_{\left(i,j\right)}^{\left(\tau |x^{\tau }\right)}}\right]}{{\left\{\sum_{i,j=1}^{m}{\left[P_{\left(i,j\right)}^{\left(k|x^{k}\right)}-\bar{P_{\left(i,j\right)}^{\left(k|x^{k}\right)}}\right]}^{2}\sum_{i,j=1}^{m}{\left[P_{\left(i,j\right)}^{\left(\tau |x^{\tau }\right)}-\bar{P_{\left(i,j\right)}^{\left(\tau |x^{\tau }\right)}}\right]}^{2}\right\}}^{\frac{1}{2}}}$$

with:

(17) $$\bar{P_{\left(i,j\right)}^{\left(k|x^{k}\right)}}=\frac{\sum_{i,j=1}^{m}P_{\left(i,j\right)}^{\left(k|x^{k}\right)}}{m}$$

(18) $$\bar{P_{\left(i,j\right)}^{\left(\tau |x^{\tau }\right)}}=\frac{\sum_{i,j=1}^{m}P_{\left(i,j\right)}^{\left(\tau |x^{\tau }\right)}}{m}$$

#### 2.2.6 CPS concept

According to Dingle and Drake [60], migration is an adaptation of spatiotemporally fluctuating resources. In the context of changepoint grouping, adaptation and resources can be assimilated by occurrence and climatic disturbances, respectively. Therefore, changepoint migration can be defined as the occurrence of a changepoint or changepoint grouping during climatic disturbances that spatiotemporally fluctuate. Therefore, in our context, the latter represents a spatiotemporal representation of the posterior probability.

We defined a signal by the posterior probability $(P_{\left(i,j\right)}^{\left(k|x^{k}\right)}$) of a changepoint or changepoint grouping, and classified its intensity interval as very weak [0.00, 0.30], weak ]0.30,0.50], strong ]0.50,0.75], or very strong ]0.75,1.00]. A signal does not exist if its intensity is extremely low. However, “the signal occurrence” is defined in the interval [0.30, 1.00]. According to this classification, the detection of a changepoint or changepoint grouping is defined as a strong or a very strong signal. In this context, the “dwell time or the lifetime of a signal” can be defined based on its occurrence. However, signal persistence is defined as a lifespan ≥3 years. If we observe signal persistence followed by its disappearance and again its persistence, the year in which the signal disappears constitutes a single persistent period for this signal. The results of the persistence of a detected change point can be validated using the signal persistence method defined in the context of migration. Analysis of different time windows makes it possible to visually determine the lifespan of a signal and estimate its persistence. The estimation of the latter can be considered an observed measurement used to validate the estimated measurement using the Pearson correlation method. The difference observed between these two measurements defines the error in estimating signal persistence from the probability estimated using Pearson's correlation method. However, the error in estimating the lifespan of a climatic disturbance is considered an absolute value, unlike the error in estimating the persistence period, which considers the direction of the changepoint axes. The positive or negative signs in front of the persistence period estimation error values represent an over- or underestimation of the CPS method compared with Pearson's correlation method, respectively. This error was represented by three components: [a, b, c]. Components a and b provide the errors of the start and end of the persistent period, respectively, whereas component c provides the lifetime error of the persistent period.

## 3 Results

### 3.1 Changepoint detection and the amount of shift

**Fig. 2:**
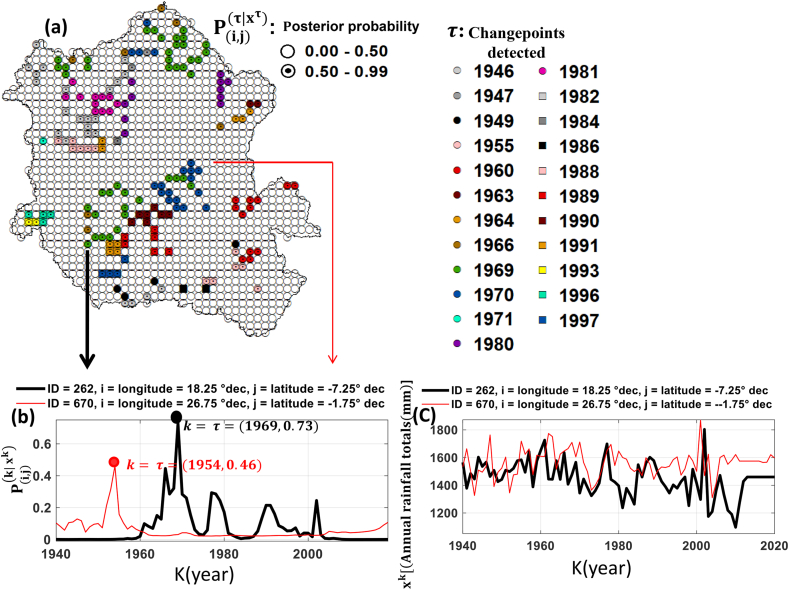
Changepoints detection in the Congo Watershed and posterior distribution of changepoint k: (a) the posterior mode of k where the ranges of values [0.00, 0.50] and ]0.50,1.00] respectively designate the detected and undetected changepoints k, (b) posterior distribution of changepoint k ($P_{\left(i,j\right)}^{\left(k|\;x^{k}\right)}$**)** and (c) annual rainfall totals ($x^{k}$**)**.

A change point is detected when the value of its posterior probability belongs to the interval [0.5, 1]. Fig. 2 presents the detected and undetected change points in the Congo Watershed (Fig. 2a). It also illustrates two examples of the posterior distribution of k (Fig. 2b) and the time series of the total annual rainfall (Fig. 2c). The most dominant point, estimated at 3.52 % of the total Congo Watershed area, was detected in 1969, both in the north and south (Fig. 2a). The second most dominant point, located north and south of the basin, was detected in 1970, followed by 1982, in the northwest. The southwest and east of the Congo Watershed were dominated by the change points detected in 1996 and 1960, respectively. However, the change points detected in the current study cover approximately 15.18 % of the total area of the Congo Watershed (Fig. 2a).

**Fig. 3:**
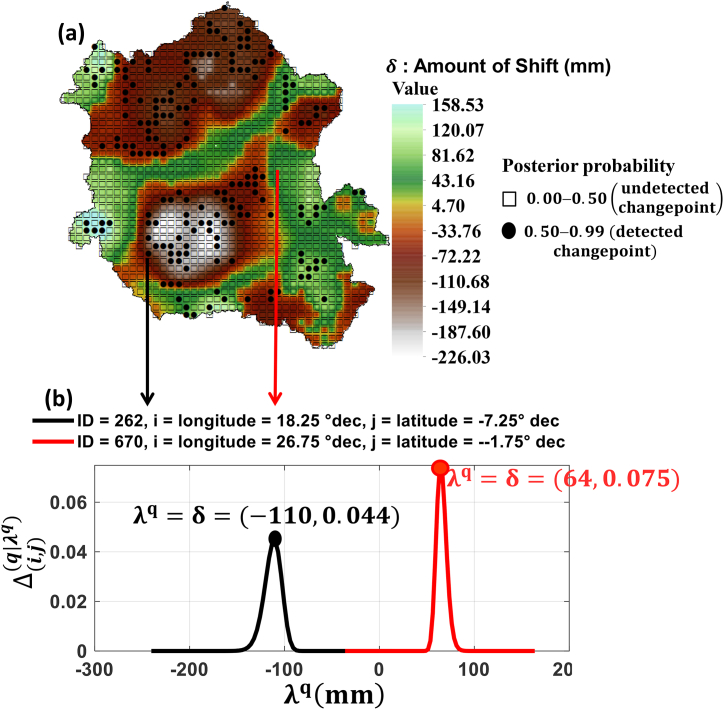
The amount of shift ($\lambda^{q}$) in the Congo Watershed and its posterior distribution ($\Delta_{\left(i,j\right)}^{\left(q|\lambda^{q}\right)}$): (a) the posterior mode of changepoint k and the amount of shift ($\lambda^{q}$) in mm, (b) posterior distribution of the amount of shift.

Fig. 3 represents the superposition of the amount of shift and the posterior mode of k on the Congo Watershed scale (Fig. 3a) and two examples of the posterior distribution of the amount of shift (Fig. 3b). As observed, the amount of shift at the changepoint reflected a decrease in precipitation in the Congo Watershed, estimated at 150.78 mm in the north and 187.45 mm in the south. Spatially, a negative shift in precipitation was detected over a large area of the Congo Basin (Fig. 3a) showing the regions characterised by a negative the amount of shift. Nevertheless, there were marginal areas westward and eastward in which precipitation increased (Fig. 3a).

A negative shift in precipitation was detected in over 60 % of the total area of the Congo Watershed. In particular, Fig. 2, Fig. 3a allow us to observe that the changepoints detected in 1969, 1970, 1981, 1982, 1988, 1989, 1990, 1991 and 1997 were all characterised by a negative the amount of shift (decrease in precipitation) and the area covered by these points is estimated at approximately 9.50 % of the total Congo Watershed area. This illustrates that during the last three decades of the past century, the Congo Watershed was the driest over our entire study period.

### 3.2 Time window selection

**Fig. 4:**
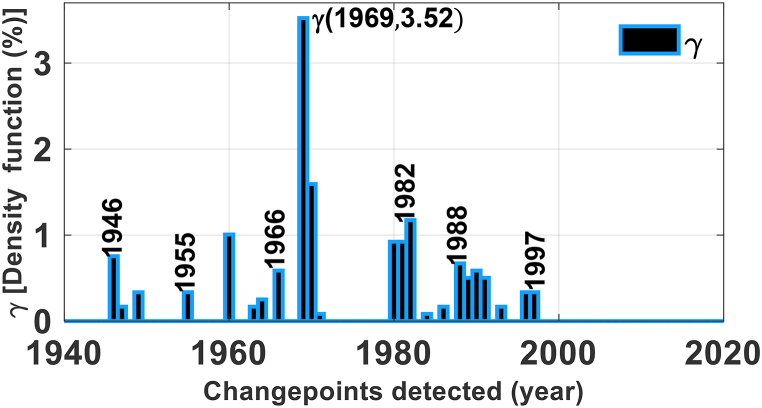
Density function of detected changepoints (**γ)** over the period from 1940 to 2020 which shows the most dominant point at the year 1969.

The density function estimation of the change points detected on the Congo Watershed scale is shown in Fig. 4. The latter shows that the maximum value of the detected change-point density corresponds to the point detected in 1969. Consequently, 1969 corresponds to the time window selected for probability spatial variability. However, the points detected in 1970, 1982, and 1960 were in decreasing order of the density function values, the most dominant after those detected in 1969 on the Congo Watershed scale (Fig. 4). These points were located in the north and south (1969 and 1970), northwest (1982), and east (1960) of the Congo Watershed (Fig. 2a) and were all characterized by a decrease in precipitation (Fig. 3a).

### 3.3 Location of changepoint grouping detected in 1969

**Fig. 5:**
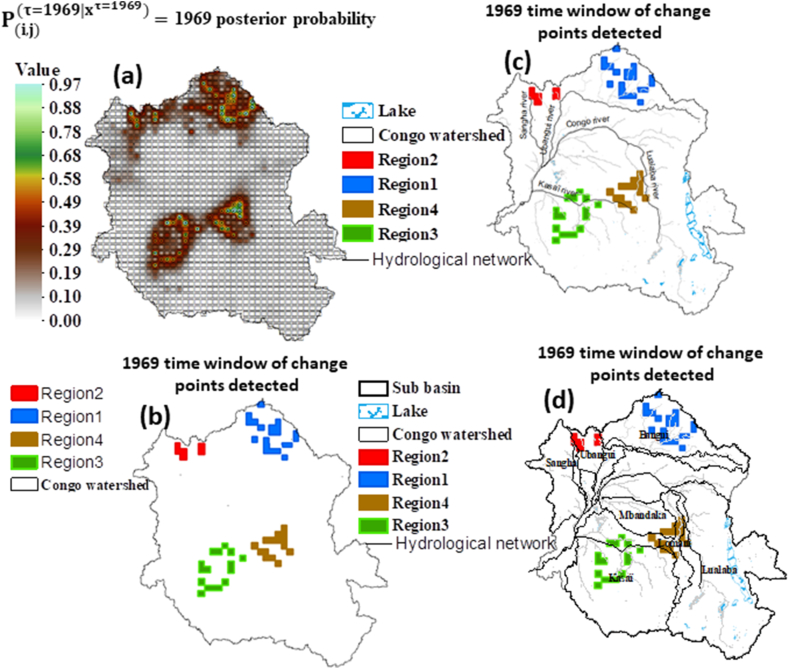
Location of changepoints grouping detected in the 1969 time window: (a) 1969 time window where $P_{\left(i,j\right)}^{\left(\tau =1969|x^{\tau =1969}\right)}$ denotes the posterior probability of changepoint τ at 1969, i, j and k respectively, longitude, latitude and time serie. (b) detected changepoints grouping, (c) location of changepoints grouping in relation to the primary tributaries of the Congo River, (d) location of changepoints grouping in relation to the Congo Watershed sub-basins.

Fig. 5 presents the 1969 time window of the posterior probability spatial variability (Fig. 5a), classifies (Fig. 5b), and locates the points detected in this time window with respect to the three primary tributaries of the Congo River (Fig. 5c) and the delimitation of different sub basins of the Congo Watershed. Fig. 5a shows the values of the posterior probability in the 1969 time window belonging to the interval [0.50, 1.00]. These values allow us to observe the groupings of points detected in the time window of 1969. The geographical locations of the detected points allowed us to classify these groupings into four regions, two each in the north and south (Fig. 5b). However, Fig. 5d clearly shows that Region 1 is entirely located in the Bangui sub basin, Region 2 is northwest of the Ubangui sub basin, and Region 3 belongs to the Kasaï sub basin.

However, a grouping of points belonging to Region 4 was located in the Kasaï sub basin and another grouping further north of Region 4 was located in the southeast of the Mbandaka (called Ruki) sub basin, roughly in the centre of the Lomani (called Lomami) sub basin and east of the Lualaba sub basin (Fig. 5d). Fig. 5c shows that the Ubangui and Kasaï Rivers were strongly impacted by the 1969 changepoint.

### 3.4 Changepoint persistence detected in 1969

**Fig. 6:**
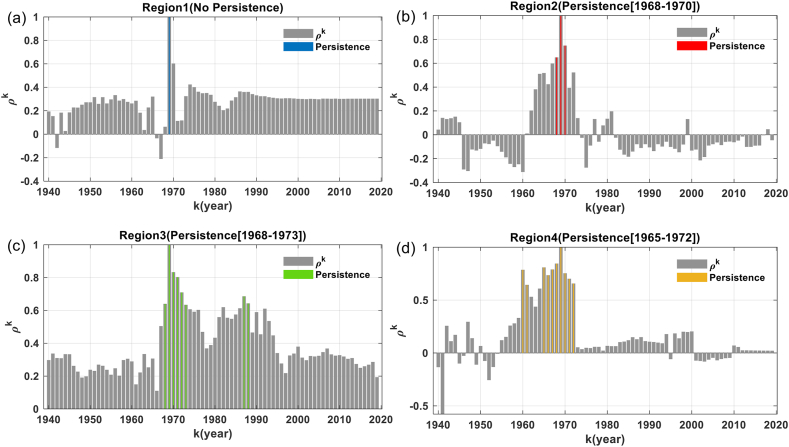
Temporal variation of Pearson's correlation coefficient ($\rho^{K}$) in the [0.6, 1.00] interval which illustrates the similarity of the posterior probability of the different time windows over the study period. The posterior probability of the 1969 time window is taken as a reference at different scales of the grouping regions of the detected changepoints in: (a) Region 1, (b) Region 2, (c) Region 3, and (d) Region 4.

Fig. 6 shows the similarity estimated using Pearson's correlation of the posterior probability values for the different time windows observed over the study period. This shows that Region 1 did not persist over the study period (Fig. 6a); Region 2 persisted from 1968 to 1970 (Fig. 6b), unlike Region 3, which persisted from 1969 to 1972 (Fig. 6c). Region 4 persisted from 1965 to 1972 (Fig. 6d), that is, a lifetime of eight years.

Table .1 summarises: the areas, persistence periods and lifetimes of different regions of the changepoints detected in the 1969 time window. It also presents the error of the persistence estimate obtained using Pearson's correlation method.

**Table 1:** Persistence period and lifespan of changepoint regional groupings detected in 1969.

Region	Persistent period and duration of persistence		Error (years)	Surface area of the region in ${\text{Km}}^{2}$ (%)
	$\rho^{k}$	Climate disturbance signal		
1	No persistence	No persistence		65057.58 (1.76 %)
2	[1968–1970,3]	[1968–1970,3]	[0, 0, 0]	18587.88 (0.50 %)
3	[1968–1973,6]	[1966–1970,5]	[-2, −3, 1]	49567.68 (1.34 %)
4	[1965–1972,8]	[1968–1971,4]	[+3,- 1, 4]	46469.70 (1.26 %)

The area, expressed as a percentage, of a region grouping the changepoints detected in 1969 is estimated in relation to the total area on the Congo Watershed. $\rho^{k}$ Pearson correlation coefficient. [lower limit of persistent period, upper limit of persistent period, duration of persistence].

Fig. S4 in the Supplementary Material presents the spatial variability of the posterior probability of the different time windows from 1943 to 2017 which makes it possible to validate the results on the regional persistence of changepoint groupings detected in 1969.

Fig. S4 shows that the occurrence of the signal of climate disturbance was observed only over the 1969 and 1970 time windows on the Region 1 scale (Figs. S4c and S4d). This means that Region 1 was not persistent over our study period. The occurrence of the signal on the Region 2 scale was observed in 1966, 1968, 1969 and 1970 (Figs. S4c and S4d) with dominant intensity values close to 0.39, 0.34, 0.60 and 0.39 respectively. Based on this observation, the signal persisted from 1968 to 1970 on the Region 2 scale thus validating the persistence estimate obtained using Pearson's correlation method (Table 1). Occurrence of the signal on the Region 3 scale was observed from 1966 to 1970 (Figs. S4c and S4d), however, we observed an expansion of the signal area from 1966 to 1969. From 1970, a considerable reduction in the intensity and expansion of the surface area of the signal was observed until 1971. The persistence of this signal was then estimated from 1966 to 1970 which makes it possible to validate the persistence of the signal estimated using Pearson's correlation with an error of [−2, −3, −1] (Table 1). On the Region 4 scale, occurrence of the signal was observed from 1968 to 1971. However, this occurrence continued in the southern part of Region 4 until 1978 (Figs. S4c and S4d) suggesting a subdivision of Region 4 into two sub-regions. The error in the estimation of the persistence of Pearson's correlation method is estimated in the interval [+3, −1, −4].

### 3.5 CPS migration

Fig. S4 also allows us to describe changepoint signal migration at the Congo Watershed scale. From 1944 to 1945, a weak signal appeared further northeast of the watershed, disappeared from 1946 to 1950, and reappeared from 1951 to 1952 with very weak intensity. This signal appeared as weak and very weak two years later, in 1953 and 1954, respectively, and then disappeared from 1955 to 1961. This signal then became strong, very strong, and weak from 1962 to 1965 and disappeared again from 1966 to 1978. Although the intensity of this signal was very strong in 1980, it remained very weak from 1979 to 1984, and disappeared again from 1985 to 1999. It follows from this observation that the signal observed further northeast of the Congo Watershed persisted from 1962 to 1965 (four years). Southeast of the Congo Watershed, another signal also appeared in 1949 and 1950 with a maximum intensity of 0.75 and 0.47 respectively, which disappeared from 1951 to 1953 and persisted from 1954 to 1956 and 1958 to 1960, although it was weakened in 1957 with a maximum intensity of 0.26, which persisted for seven years (from 1954 to 1960). Another signal that persisted from 1958 to 1961 was observed in the central-eastern region of the basin.

Further north-westward, we observed signal persistence from 1946 to 1948. To the south of Region 2, northwest of the Congo Watershed, another signal appeared in 1980, which persisted until 1991 (11 years). The intensity of this signal decreased over time. Further southwestward of the Congo Watershed, another signal also appeared which was very weak from 1982 to 1987, although it persisted from 1992 to 1997.

South of Region 3, another signal persisted from 1989 to 1992. Although a signal was observed southeastward of the Congo Watershed, it did not persist.

Table .1 presents the persistence of other signals that are not described above and Table .2 summarises the persistence of different signals described above, including the persistence of the signals in Table .1.

**Table 2:** Persistence period and maximum CPS intensity on the Congo Watershed scale.

East	Number of signals	Maximum signal Intensity (year)	Persistence (period [year])
North	1	0.98 (1963)	1962–1965 [4]
central	1	0.96 (1960)	1958–1961 [4]
South	1	0.96 (1960)	1954–1960 [7]
**Central**	**Number of signals**	**maximum intensity (year)**	**Persistance**

North	2	0.99 (1969)	Non-persistent signal
		0.99 (1969)	1968–1970 [3]
central	0	No intensity	No signals
South	3	0.99 (1969)	1966–1970 [5]
		0.99 (1969)	1968–1971 [4]
		0.99 (1990)	1989–1992 [4]
**West**	**Number of signals**	**maximum intensity (year)**	**Persistance**

North	2	0.98 (1946)	1946–1948 [3]
		0.98 (1981)	1980–1991 [12]
central	0	No intensity	No signals
South	1	0.75 (1996)	1992–1997 [6]

Based on the maximum intensity of the CPS or the grouping of the detected change points presented in Table .2, we can describe the migration of these signals. As detected in the northwest of the Congo Watershed in 1946 (Table 1), the signal migrated east of the basin, with an average duration of 15 years (until 1960). From the latter, it continued its migration towards the central basin for an average duration of nine years (until 1969), and then migrated again towards the northwest of the Congo Watershed for a period of 12 years. In the northwest in 1981, the signal descended towards the south of the Congo Watershed after a period of nine years (in 1990) and remained there until 1997. However, migration of the signal from 1946 to 1960 can be reduced to 13 years if we consider the persistence of this signal from 1948 to 1960, although 1948 had a very low intensity compared with that of the first two years. Therefore, we can estimate average signal migration duration of 10 years and 8 months at the basin scale. Unfortunately, the remaining years (1938, 2001, and 2011) of this CPS migratory cycle were not captured due to low sampling of observation stations from 1901 to 1943 and from 1990 to 2020 (see Supplementary Material).

Fig. S4 clearly shows that the hydrological system of the Lualaba River was strongly affected by the climate during the 1950s. From the 1960s, disturbances affected the drainage system of the Ubangui River, until the early 1970s. In the latter, the hydrological systems of the Lualaba and Kasaï Rivers, in particular throughout the 1970s, were strongly affected by disturbance. However, at the beginning of the 1980s, disturbances affected the hydrological systems of the Ubangui River, including its primary tributaries. During the 1980s, this disturbance directly affected the Congo River (Fig. S4). The end of the 1980s and the beginning of the 1990s were characterized by the disruption of the Kasaï River hydrological system. The 1990s were marked by the disruption of a small zone in the city of Kinshasa, unlike the entire basin that was not disrupted. At the beginning of the twenty-first century, this disturbance did not affect the primary tributaries or the Congo River but was localized south of the Lualaba hydrological system. The analysis of the CPS on the decadal time steps illustrated in Fig. S4 clearly shows a 10 year cycle. Throughout the cyclical course of this signal, different hydrological systems were affected in turn at 10-year intervals.

## 4 Discussions

We detected changes in the precipitation time series in the Congo Basin and their amount of shift, spatial range, and persistence. The results of this study highlight the changes in precipitation during 1946–1997 (Fig. 2, Fig. 3, Fig. 4) over a large and significant portion of the Congo Basin. However, 1969 was characterized by a greater extent of areas affected by the change points (Fig. 4), geographically concentrated in two sub basins: Ubangui and Kasaï (Fig. 5). The change points from 1969 until the end of the twentieth century were all characterized by a reduction in precipitation with a maximum magnitude of 226.03 mm (Fig. 3a). Simultaneously, rainfall increased in some of the eastern and western regions of the basin, with a maximum magnitude of 158.53 mm (Fig. 3a). These changes persisted, on average, over five years (Table .2) leading to a persistent drought that made the Congo Watershed slightly drier during the last three decades of the last century, as noted by Ndehedehe et al. [6]. There are several possible causes for discontinuities in climate-related time series [61]. Nevertheless, if we consider the dates of the locations, spatial ranges, and persistence of change points, we can suggest that natural variability (weather pattern shift) contributed significantly to the reduction in precipitation over the 1946–1997 period of severe climatic disturbances (Fig. 4).

Several studies in West and Central Africa [[10], [11], [12], [13], [14], [15],^21^,[24], [25], [26]] have highlighted the response of surface and groundwater resources to climate variability. However, all agree that the date of 1969 or 1970 (Fig. 4) corresponds to a significant major event in climatic variability. However, none of these studies (Wesselink et al. [25], Orange et al. [24], Nguimalet and Orange [26], Laraque et al. [10,11], Bogning et al. [21], and Moukandi N'kaya et al. [12]) estimated the total area affected by this climatic event in 1969 or 1970 at the Congo Watershed scale. This area is estimated to constitute 3.5 % of the Congo Watershed. However, over the last three decades of the last century (1969–1997), we estimated that 9.5 % of the Congo Watershed has been affected by drought. According to Ndehedehe et al. [6], the period from 1969 to 1997 was devoid of extreme drought episodes, thus justifying the estimation of this surface area being located primarily in the north and south of the Congo Watershed (Fig. 3). This underlines the regional nature of the highly significant spatiotemporal heterogeneity of climate disturbances.

The drainage systems of the Congo River have not spared from this drought [12], as demonstrated by the navigability problems experienced along the Ubangui [31] and Kasaï Rivers [32]. In 1975, Pandi et al. [31] observed a reduction in the number of navigable days along the Ubangui River as indicated by the appearance of large sandbanks. Along the Kasaï River, Kisangala [32] observed a drop in the water level with direct repercussions on navigability through the emergence of rocks and sandbanks. Despite its role in attenuating low water levels in the Congo Watershed [62], Cuvette Centrale has not been spared from this drought [21] over the last three decades of the last century (Fig. 3), as demonstrated by Zhou et al. [2].

Although the three primary tributaries of the Congo Watershed drainage system were identified as being affected by climatic disturbances in 1969 and 1970 [12], this study showed that the primary tributary of the Ubangui drainage system, the Uélé River, was unaffected. The latter only affected the secondary tributaries of this system (Fig. 5). In contrast, the Sangha River drainage system is affected upstream (Fig. 5), as reported by N'kaya et al. [12]. The latter detected a change point in 1971, from 1950 to 1999, in the Sangha sub basin in Ouesso using Pettitt's [36] method. The climatic disturbances of 1969 and 1970 also affected the entire Kasaï River drainage system (Fig. 5), consolidating the observations of Moukandi N'kaya et al. [12].

Over the entire observational period (1940–2020), 1946–1997 was characterized by severe climatic disturbances (Fig. 4). This suggests that climatic disturbances in the Congo Watershed persisted throughout the observational period for half a century. However, the definition of a disturbance proposed by Yi and Jackson [63], which refers to the difference between disturbance and perturbation, suggests that this transitional period is characterized by intense positive and negative feedback. Furthermore, the recent wetter hydroclimatic conditions after this transitional period [64,65] led us to suggest resilience [63] of the Congo Watershed climatic system in response to this disturbance. In view of this suggestion, this disturbance can be considered minor [66]. This observation supports the theory of transient phenomena [67]. According to this theory, a system can persist for an extremely long transient period (several years or decades), characterized by a long-term stable dynamic regime (quasi-constant, cyclic, or even chaotic) that is not a long-term stable regime that would eventually occur. These long-term persistent transients can provide an alternative explanation for regime shifts in a system [67]. This can lead to catastrophic changes in the structure and function of the system, including species extinction and biodiversity loss [[68], [69], [70]]. This loss has also been observed in the northern tropical forests of the Congo Watershed [2].

However, this transitional period (1946–1997), which affected 15 % of the total Congo Watershed area, was also devoid of extreme and persistent droughts, unlike the other two periods (1901–1920 and 1990–2010) [6]. This suggests that, with the exception of this transitional period, the Congo Watershed experienced only episodes of extreme and persistent drought [6]. Consequently, this apparently wet watershed is dry. Our results suggest that 60 % of the total surface area of the Congo Watershed is affected by drought (Fig. 3). However, it should be noted that the Kasaï sub basin was the driest, unlike the Ubangui sub basin (Fig. 3). Ndehedehe et al. [7] highlighted the extent of drought in the southern basin. Nevertheless, the western and eastern parts of the Congo Watershed are generally wettest (Fig. 3), as noted by Laraque et al. [11].

Although the climatic disturbances of 1969 and 1970 did not persist at the Ubangui sub basin scale, considering the very weak signal of climatic disturbance, this persistence could be estimated at eight years (Fig. S4). Therefore, redefining the CPS intensity intervals as [0.00,0.1], ]0.1,0.50], ]0.50,0.75] and ]0.75,1.00], as very weak, weak, strong, and very strong, respectively, would most likely lead to a good estimate of disturbance persistence. In addition, considering the reduction in the Pearson's correlation coefficient threshold value (for example, to 0.5) leads to a considerable reduction in the persistence estimation error. However, it should be noted that the effects of climatic disturbance in 1969 and 1970 on the environment of the Congo Watershed lasted for an average of five years (Table 1).

However, the lack of persistence of the climatic disturbance of 1969 in the Ubangui sub basin can be attributed to the method and length of the time series used [12,71,72]. Two time series with the same variables and sources but different lengths of the same hydrological unit can lead to different results for the location of a disturbance [12,72]. In contrast, Pettit's [36] method was found to be robust [71], more efficient [33,71], highly sensitive to the length of a time series [72], and detected a much higher number of disturbances in a data sample [71] than Lee and Heghinian's [37] method.

Climatic disturbances were not observed in 1982 and 1991–1992 in the hydrological systems of the Ubangui [26] and Kasaï Rivers [12], respectively however, the current study clearly shows that on both dates, both hydrological systems were affected by the disturbance (Fig. S4). This shows that the dates of climatic disturbances coincided with the dates of the hydrological droughts. Furthermore, the reduction in surface runoff in the Ubangui and Kasaï River hydrological systems every ten years can be explained by the migration of the climatic disturbance signal every ten years (Table .2). Consequently, the 10-year cyclic dynamics of the CPS during the 1946–1997 transitional period were responsible for the 10-yearly reduction in hydrological drought observed in the Ubangui and Kasaï River hydrological systems. This cycle was also highlighted by Kazadi and Kaoru [73] for 1960–1992, which was located during the transitional period 1946–1997.

However, because of the monthly scale involved in the propagation time from meteorological to hydrological drought [[74], [75], [76], [77]], the 8-month error in this cycle was considered to be associated with the propagation time. This suggests that during the transitional period (1946–1997), the propagation time from climatic disturbances to the occurrence of hydrological drought in the Congo Watershed averaged 8-months.

This study provides a simple tool for delineating and monitoring areas affected by climatic disturbances, using a grid database. However, this tool depends on the threshold value set by a posteriori probability of the changepoint, resolution, and quality of the database, and the statistical method used. Nevertheless, the tool allows precise visualization of the locations of surfaces affected by change points, unlike some approaches that predefine and delineate these spaces upstream.

## 5 Conclusion

This study improves our understanding of hydrological responses to disturbances and climatic variability by identifying the cyclic dynamics of a lengthy transient process as a key factor in explaining the reduction in surface runoff every 10 years.

During our observational period (1940–2020), the climate of the Congo Watershed was severely disrupted from 1946 to 1997. This period of climatic disruption was characterized by a cyclical dynamic of approximately 10 years and 8 months. During this transition, the Congo hydrological systems and watershed ecosystems were modulated by cyclical dynamics. However, because of the monthly scale involved in the propagation time from meteorological to hydrological drought, the eight-month error associated with this cycle was considered to be the propagation time from climatic disturbances to drought or humidification. Therefore, we recommend research focused on estimating the propagation time in the Congo Watershed to verify this hypothesis. The wavelet method is an excellent method for quantifying this cycle. Nevertheless, recent observations of wetter hydroclimatic conditions suggest that a small disturbance is associated with this lengthy transitional period, underlining the resilience of the basin climatic system.

Our qualitative analysis provides proof of the signature of climate dynamics on drought hydrological dynamics. This study provides a decision-making framework for water resource managers and for the proper operation of water retention and catchment projects by proposing a simple tool for delineating climatic zones that can be used to identify ecosystems affected by drought or flooding.

## CRediT authorship contribution statement

**Louis Kongoda Lisika:** Writing – review & editing, Writing – original draft, Visualization, Validation, Supervision, Software, Project administration, Methodology, Investigation, Formal analysis, Data curation, Conceptualization. **Jacques Celestin Moliba Bankanza:** Writing – review & editing, Writing – original draft, Visualization, Validation, Resources, Methodology, Funding acquisition, Formal analysis, Conceptualization. **Louis Efoto Eale:** Writing – review & editing, Writing – original draft, Visualization, Validation, Resources, Methodology, Formal analysis, Conceptualization. **Petrus Bompere Lemo:** Writing – review & editing, Writing – original draft, Visualization, Validation, Software, Resources, Formal analysis, Data curation, Conceptualization. **Jean Kigotsi Kasereka:** Writing – review & editing, Writing – original draft, Visualization, Validation. **Jean-Robert Bwangoy Bankanza:** Writing – review & editing, Writing – original draft, Resources, Investigation, Funding acquisition. **Vincent Lukanda Mwamba:** Writing – review & editing, Writing – original draft, Validation, Supervision, Project administration, Investigation, Formal analysis.

## Declaration of competing interest

The authors declare that they have no known competing financial interests or personal relationships that could have appeared to influence the work reported in this paper.
