# Supplementary material for: Signature of climate dynamics on hydrological drought dynamics: A qualitative analysis
Source: Heliyon. 2024 Oct 26;10(21):e39822. doi: 10.1016/j.heliyon.2024.e39822 (PMC11550595; doi:10.1016/j.heliyon.2024.e39822)
Supplement: Multimedia component 1 [file mmc1.docx]

**Title: Insufficient data over the period from 1991 to 2020 in the CRU T.S 4.05 database at the Congo watershed scale and illustration of the low intensity of the climate change signal over the period from 1999 to 2020.**

The Insufficient data in a region is an obstacle to hydro-climatic studies. The Congo watershed is one such ungauged region [1] [2]. Even though a few rainfall databases exist for this catchment area, it seems very important, given the lack of data in this catchment area, to carry out an analysis of the number of rain gauge density used by these databases, in particular the CRU TS 4.05 database. This analysis will enable us to pinpoint periods and regions with high or low number of rain gauge densities.

The CRU TS 4.05 database not only provides data on climatic parameters, it also makes available to researchers the number of rain gauges used in the interpolation of the value of an elementary mesh of 0.5° x 0.5° resolution. For this purpose, let Stn denote the number of rain gauge per 0.5° x 0.5° grid element on the CRU TS 4.05 grid.

Fig.S.1a shows the monthly variation of Stn≥1 and Fig.S.1b illustrates the annual minimum monthly variation of Stn≥1 noted by StnMin. Fig.S.1a shows that Stn≥1 began to increase from January 1901 until it reached its maximum value in January 1937. From the latter, the area of Stn≥1 was characterized by a perfectly linear curve until December 1989 (Fig.S.1a). The period from January 1990 to December 2020 is characterized by a decreasing curve. Three periods of Stn≥1 variability follow from this observation. The first extends from January 1901 to January 1937, the second, which is dense in data, covers a period from 02/1937- 12/1989, and the last, which is characterized by a decrease in Stn≥1, covers the period 01/1990-12/2020. We observe that the StnMin curve follows the same pattern as that of Stn≥1, however, the data-dense period is observed from 1931 to 1991.


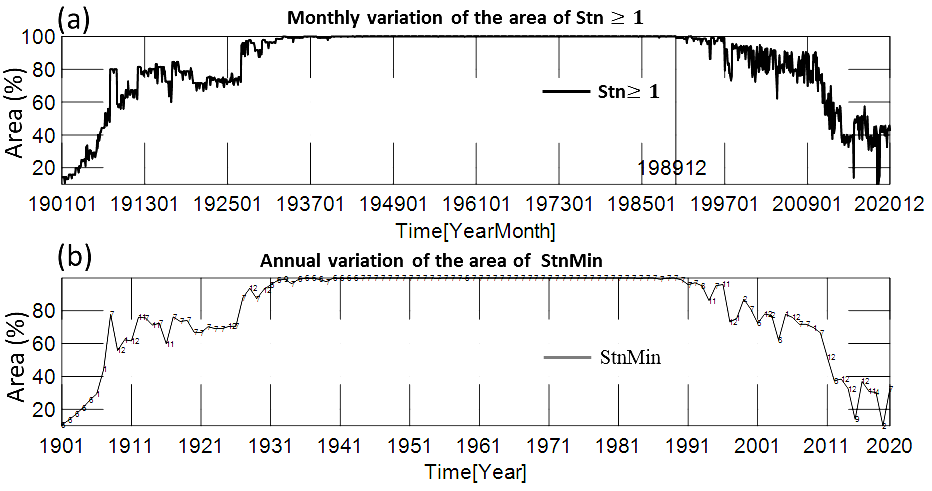


**Fig.S.1 (a)** Monthly variation in the area of the number of rain gauge greater than or equal to 1 per elementary grid of 0.5°x 0.5° (Stn≥1) expressed as a percentage at the Congo watershed scale and (**b)** annual variation in monthly minimum of Stn≥1 noted by StnMin expressed as a percentage at the Congo watershed scale.

The spatio-temporal variation of Stn≥1 shown in Fig.S.2 highlights the decrease over time in number of rain gauge density observed mainly towards the east of the Congo watershed, characterized by white-colored areas indicating a zero number of rainfall gauges in an elementary grid.

Generally speaking, the western part of the basin shows high Stn≥1 values between 5-8 over the study period. Although the central part of the basin appears to have Stn≥1 values between 1-3, it is however attenuated by areas with white coloration. The 2012-2017 period is characterized by a virtual absence of the number of rain gauge in the eastern part of the basin.


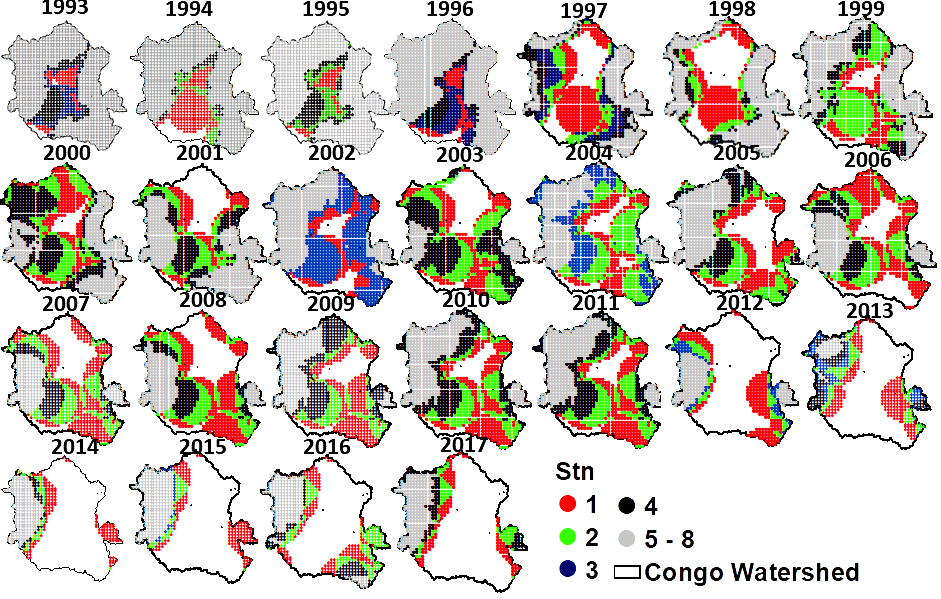


**Fig.S.2**.Spatio-temporal variability in the number of rain gauge greater than or equal to 1 per elementary grid of 0.5°x 0.5° (Stn≥1) from 1993 to 2017 on the Congo watershed scale.

Fig.S.3, which shows the monthly variation of each Stn value, shows that the data-dense period is observed from July 1943 to February 1990 (Fig.S.3a and Fig.S.3i). We observe that Stn gauge areas do not exceed 50% for all Stn values except 0 and 8. The lowest percentage is observed for Stn = 7 (Fig.S.3h).


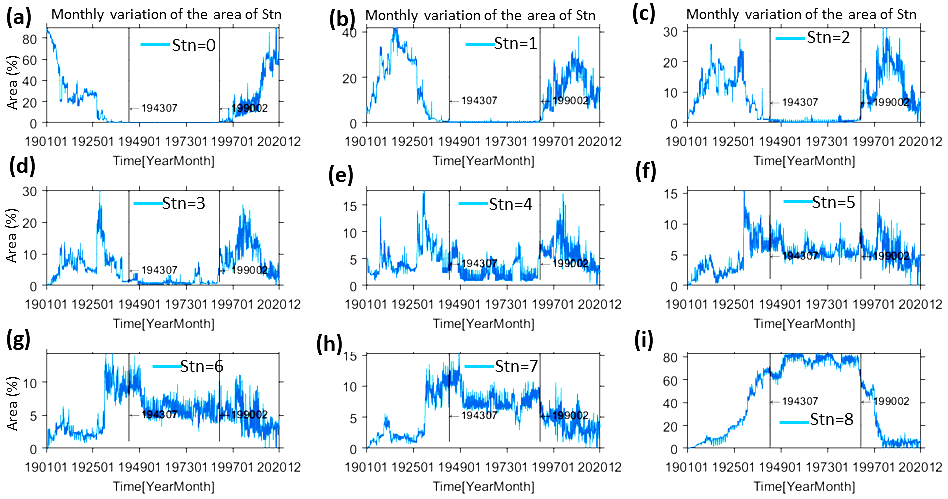


**Fig.S.3**.Monthly variation of each Stn value : **(a)** Stn=0, **(b)** Stn=1, **(c)** Stn=2, **(d)** Stn=3, **(e)** Stn=4, **(f)** Stn=5, **(g)** Stn=6, **(h)** Stn=7, **(i)** Stn=8. Stn denote the number of rain gauge per 0.5° x 0.5° grid element on the CRU TS 4.05 grid.

**
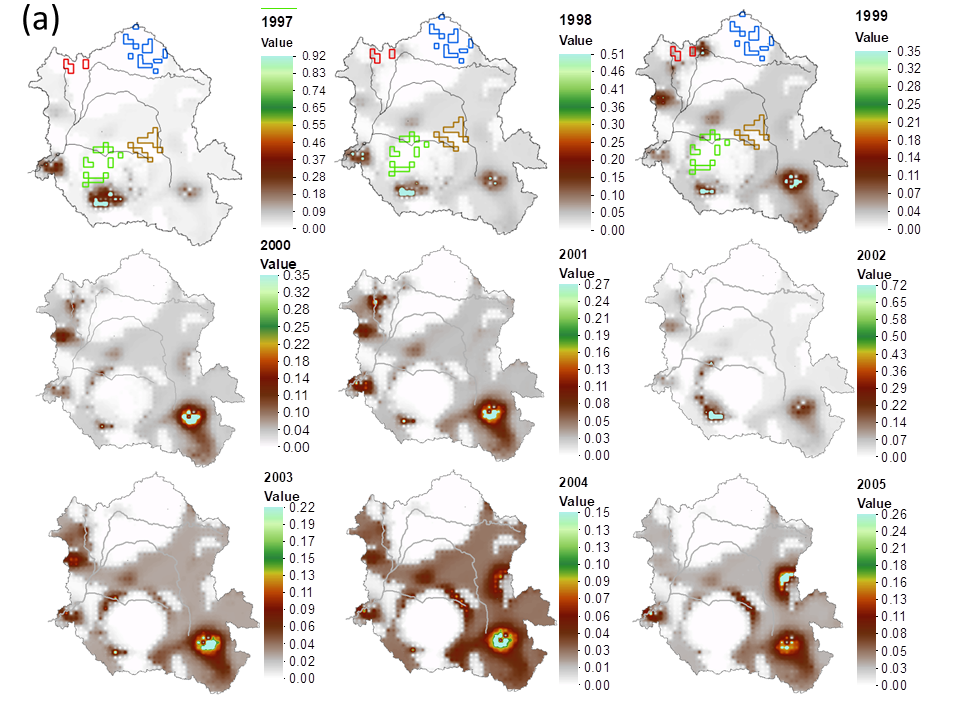
**

**
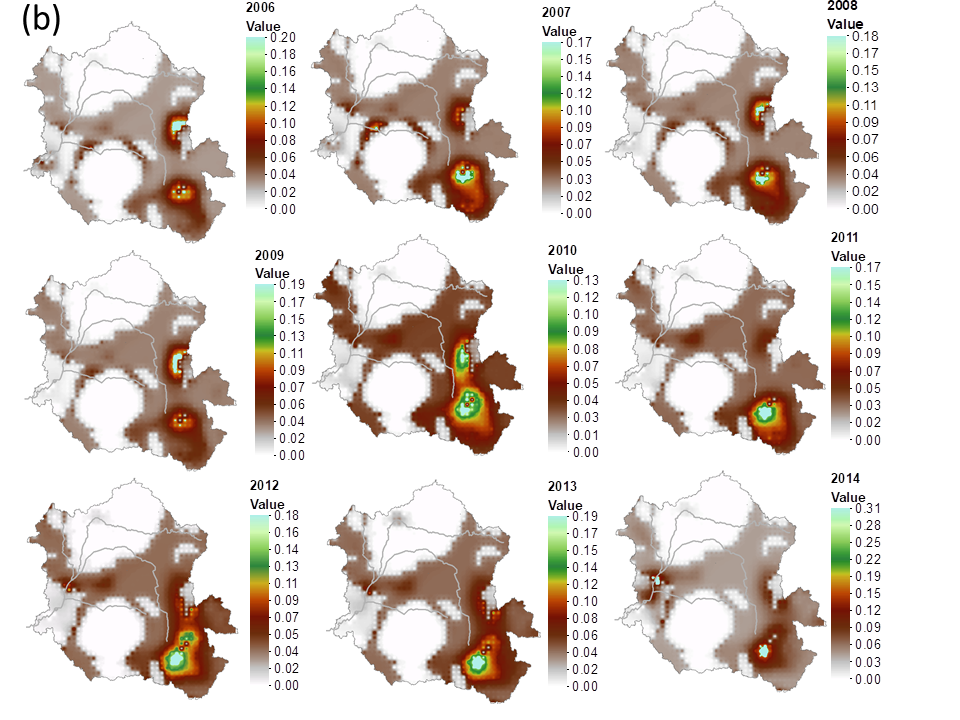
**

**
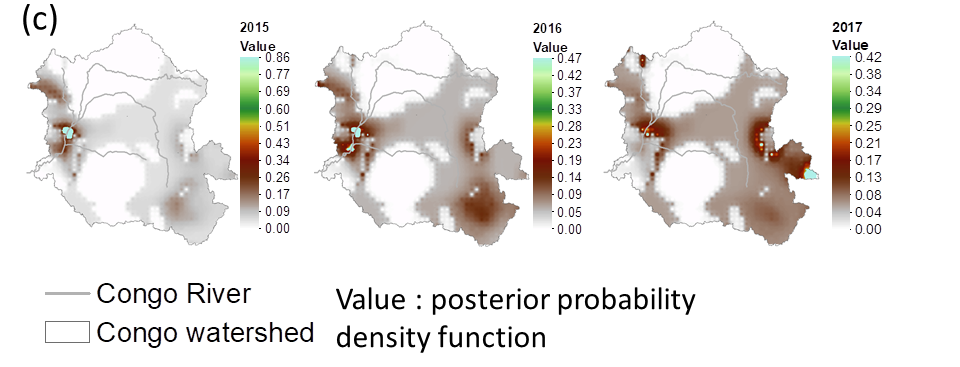
**

**Fig.S.4**.The posterior probability from 1997 to 2017: (a) 1997 to 2005, (b) 2006 to 2014, (c) 2015 to 2017.

**References**

1. A. Laraque, G. D. Moukandi N'kaya, D. Orange, R. Tshimanga, J. M. Tshitenge, G. Mahé, C. Nguimalet, M. Trigg, Y. 8Identifi, S. Yepez et G. Gulemvuga, Recent Budget of Hydroclimatology and Hydrosedimentology of the Congo River in Central Africa, *Water.* 12(2020) 2613. https://doi.org/10.3390/w12092613.
2. R. Washington, R. James, H. Pearce, W. M. Pokam, W. Moufouma-Okia, Congo Basin rainfall climatology: can we believe the climate models?, Phil. Trans. R. Soc. B. 368(2013) 20120296.https://doi.org/10.1098/rstb.2012.0296.
